# Pulmonary Artery Stiffness by Cardiac Magnetic Resonance Imaging Predicts Major Adverse Cardiovascular Events in patients with Chronic Obstructive Pulmonary Disease

**DOI:** 10.1038/s41598-018-32784-6

**Published:** 2018-09-27

**Authors:** Lucia Agoston-Coldea, Silvia Lupu, Teodora Mocan

**Affiliations:** 1 2nd Department of Internal Medicine, Division of Cardiology, Iuliu Hatieganu University of Medicine and Pharmacy, Cluj-Napoca, Romania; 2 5th Department of Internal Medicine, University of Medicine and Pharmacy of Tirgu Mures, Tirgu Mures, Romania; 3 Department of Physiology, Iuliu Hatieganu University of Medicine and Pharmacy, Cluj-Napoca, Romania

## Abstract

In this prospective pilot study, we aimed to evaluate the ability of cardiac magnetic resonance imaging (CMR) parameters of right ventricular function and pulmonary artery stiffness to identify pulmonary hypertension (PH), predict major adverse cardiovascular events (MACEs) in patients with secondary PH due to chronic obstructive pulmonary disease (COPD), and to estimate a prospective sample size necessary for a reliable power of the study. Thirty consecutive patients with COPD and suspected secondary PH were assessed by clinical examination, the six minute walk test, echocardiography, right heart catheterization and CMR, and followed–up for a mean period of 16 months to identify MACEs (cardiac death, ventricular tachyarrhythmia, and heart failure). Among CMR parameters of pulmonary artery stiffness, pulse wave velocity (PWV) yielded the best sensitivity (93.5%) and specificity (92.8%) for identifying PH, as diagnosed by cardiac catheterization. Moreover, PWV proved to be a valuable predictor of MACEs (HR = 4.75, 95% CI 1.00 to 22.59, p = 0.03). In conclusion, PWV by phase-contrast CMR can accurately identify PH in patients with COPD and may help stratify prognosis.

## Introduction

Chronic obstructive pulmonary disease (COPD) has already been acknowledged as a triggering factor for right heart failure and pulmonary hypertension (PH), due to its impact on right ventricular (RV) function and morphology, and on pulmonary artery (PA) remodelling^1^. Risk factors such as advanced age and active smoking are often associated with inflammation^2^, involving neurohumoral and metabolic changes that contribute to the development of both COPD and heart failure^3^. The estimated prevalence of heart failure in COPD is >20%^4^, higher than in age-matched controls with similar cardiovascular risk profiles^5^. PH is a common occurrence in COPD patients, and is associated with poor exercise capacity and higher mortality rates, which are not exclusively justified by lung impairment itself ^6,7^. More severe COPD is associated with a higher incidence of PH^8^, occurring in >50% of the patients who are enlisted for lung transplantation^9^. Data regarding the real incidence of mild to moderate COPD, however, are scarce, as large epidemiological trials have not been performed. The pattern and velocity of blood flow in the pulmonary circulation are both altered in PH^10^, as a consequence of decreased vascular compliance and increased PA stiffness, leading to increased resistance and additional wall stress^11^. Increased PA stiffness is associated with elevated afterload, exerting stress on the RV which first adapts by hypertrophy, then dilates, resulting in right heart failure^12^. As PA stiffness develops, dilatation also occurs and PA compliance decreases. As shown by previous studies, PA dilatation is associated with poor outcome in patients with COPD, since a PA: ascending aorta (AA) ratio >1 was shown to independently predict future acute exacerbations, diminished exercise capacity, clinically significant PH and mortality^13–15^. Invasive hemodynamic assessment of PA wall stiffness by right heart catheterization (RHC) is currently the standard for diagnosis, prognosis assessment and follow-up after treatment in PH patients. In several studies, invasively measured pulmonary artery pressure (PAP) was shown to associate with increased mortality^16^. Non-invasive methods, such as echocardiography^17^ and, more recently, cardiac magnetic resonance imaging (CMR)^10,18,19^ were used for PH assessment in combination with invasive modalities, leading to promising results. However, echocardiography can only be used for assessing the probability of PH, and studies that would endorse the use of CMR alone are still scarce.

On these considerations, we aimed to conduct a prospective study that allowed us to evaluate RV function and relationship between PA stiffness indices using CMR and adverse long-term outcome in patients with COPD and suspected secondary PH.

## Methods

### Patient selection and study design

We conducted a prospective pilot study in which we included 30 consecutive patients previously diagnosed with COPD and suspected of secondary PH (test group) who were hospitalized for acute respiratory failure in the 2^nd^ Department of Internal Medicine in Cluj-Napoca between October 2011 and October 2016.

Patients were included in the study provided they fulfilled the following criteria: (1) confirmed diagnosis of COPD, according to the criteria of the Global Initiative for Chronic Obstructive Lung Disease^20^; (2) confirmed diagnosis of acute exacerbation defined as aggravated dyspnoea, cough, or sputum production warranting a change in therapy; 3) high probability of PH by echocardiographic criteria (tricuspid regurgitation velocities >3.4 m/s); 4) absence of other factors that might have induced PH (e.g. pulmonary embolism, significant valvular heart disease, known coronary heart disease and left cardiomyopathies). Exclusion criteria were: 1) life expectancy of less than a year due to PH or other diseases; 2) contraindications for CMR (incompatible implanted devices, severe renal impairment, severe obesity); 3) time interval between echocardiography, RHC and CMR > 10 days. Each enrolled patient was submitted to a standard investigation protocol which included history taking, physical examination, chest radiographs, pulmonary function tests and arterial blood gases, echocardiography, six minutes walk test, and Holter ECG monitoring for 24 hours. All tests were performed within 10 days after admission, provided that the patients had been stabilized by standard therapy with bronchodilators (β2 agonists, n = 22; phosphodiesterase inhibitors, n = 7; tiotropium, n = 15), short courses of oral or intravenous corticosteroids, n = 29; oxygen therapy, n = 8; antibiotics, n = 17; and diuretics, n = 11). The cardiovascular risk profile was established in each case, based on personal and family history, laboratory findings and known exposure to toxins (active and passive smoking, other professional or environmental toxins). The investigators analysing CMRs were blinded to RHC results.

The test group was compared against a control group of 30 healthy volunteers, matched for age and sex, who were submitted to a query excluding any history of chronic disease, any current symptoms or ongoing treatment for a chronic ailment, with normal clinical examination and normal electrocardiogram, chest radiographs, pulmonary function tests, echocardiography, Holter ECG monitoring for 24 hours and CMR. The healthy volunteers were enrolled within the same time span as test group patients.

The study was approved by the Ethics Committee of the Iuliu Hatieganu University of Medicine and Pharmacy, Cluj-Napoca and conducted in accordance with the Declaration of Helsinki. All patients and healthy volunteers signed an informed consent at enrolment.

All data have been collected by and are the intellectual property of the authors.

Clinical follow-up was accomplished by clinical evaluation during hospital visits, telephone house-calls, or both at six month intervals until October 2016. In order to analyse event-free survival, we recorded the first occurring event from the following: death or aborted death from cardiac cause, sustained ventricular tachyarrhythmia, or hospitalization for heart failure. Hospitalizations due to non-cardiac causes were not considered significant events.

### Pulmonary function measurements

Pulmonary function was assessed using a water-sealed Collin Survey II volume displacement spirometer (Collins Medical, Inc.). Forced expiratory volume in 1 second (FEV1) and forced vital capacity (FVC) were measured with the purpose of diagnosing and grading COPD. Persistent airflow limitation and COPD were diagnosed in the presence of a FEV1/FVC ratio of less than 0.70. COPD was graded as mild (FEV1 >80% of the normal predicted value), moderate (50–79%), severe (30–49%), and very severe (<30%)^20^.

### Echocardiography

All images were acquired on an Aloka ProSound 5500 (Aloka Co. Ltd, Tokyo, Japan) echocardiograph with a wide-angle phased-array 2.5 MHz transducer. Measurements were performed according to previously published guidelines^21^. Images were recorded and analysed off-line to accurately quantify left ventricular (LV) and RV systolic and diastolic function. LV ejection fraction was calculated based on Simpson’s modified single plane method from the apical 4-chamber view and considered normal if ≥55%. Systolic PAP was calculated as the sum between the RV-right atrium (RA) gradient, as measured on the tricuspid regurgitation flow, and the RA pressure; the latter was estimated by measuring the inferior vena cava diameter and collapse with a sniff. The probability of PH by echocardiography was considered high for tricuspid regurgitation velocities >3.4 m/sec^21^.

### Right heart catheterization

RHC was performed to measure RA pressure, systolic, diastolic and mean PAP, pulmonary capillary wedge pressure (PCWP), and cardiac index, assessed by the thermodilution method. The pulmonary vascular resistance (PVR) index was calculated as the difference between mean PAP and mean PCWP divided by the cardiac output index in Wood units. PH was considered present if mean PAP ≥ 25 mmHg^22^.

### Cardiac magnetic resonance imaging

#### Image Acquisition

CMR ECG-gated images were acquired during apnoea with a 1.5-Tesla magnet (Magnetom Symphony, Siemens Medical Solutions, Germany) using a dedicated surface coil for radio frequency signal detection. Patients were required to hyperventilate briefly, then exhale and hold their breaths during image acquisition.

Firstly, standard localized views and contiguous short-axis and 4-chamber cine views covering both ventricles from base to apex were obtained by standard steady-state free precession (SSFP) cine CMR sequences in order to evaluate ventricular volumes and function. Pre-contrast imaging parameters were recorded by a standard protocol: repetition time/echo time 3.6/1.8 ms; flip angle 60°; slice thickness 6 mm; field of view 360 × 360 mm^2^; matrix 192 × 192 pixels; voxel size 1.9 × 1.9 × 6 mm; 25–40 ms temporal resolution reconstructed to 25 cardiac phases. Standard late gadolinium enhancement (LGE) images were acquired 10 minutes after the intravenous injection of a contrast agent (Gadoterated imeglumine or Dotarem, Guerbet) - 0.2 mmol/kg. Images were acquired in long and short axis views using a segmented inversion-recovery gradient-echo sequence after adjusting inversion time to optimize nulling of apparently normal myocardium. Brachial systolic and diastolic blood pressures were measured during the CMR SSFP acquisitions.

Phase-contrast sequence acquisition at the level of the main PA was made using a SSFP sequence of the RV outflow tract following an orthogonal plane to optimally visualise the main PA and pulmonary valve, provided that the imaging plane remained between the pulmonary valve and the PA bifurcation for the entire duration of the cardiac cycle^23^. To record PA stiffness parameters, the following specifications were applied to a free-breathing phase-contrast sequence: repetition time/echo time 7/5 ms; flip angle 15°; slice thickness 6 mm; field of view 360 × 360 mm^2^; matrix 256 × 256 pixels; voxel size 2.2 × 1.8 × 6 mm; <35 ms temporal resolution; 20 reconstructed cardiac phases; velocity encoding 150 cm/sec^10^. For the free-breathing sequences, the average of 2 measurements was used

#### Image Analysis

Cine and phase contrast images were evaluated by two experienced observers (one cardiologist and one radiologist) blinded to all clinical data, using specialized software (Argus, Siemens Healthineers Global).

#### Ventricular quantification

Endocardial and epicardial borders were traced semi-automatically on short-axis cine images covering the entire LV and RV in order to measure end-diastolic and end-systolic LV and RV volumes, ejection fractions and end-diastolic LV mass. End-diastolic LV and RV mass to volume ratios were calculated and used to assess concentric remodelling. The maximum left atrium (LA) and RA areas were measured in all patients from the 4-chamber view. All volumes were indexed to body surface area. Tricuspid annular plane systolic excursion (TAPSE) was measured from the mid-four-chamber cardiac view to assess RV longitudinal motion. RV remodelling was defined as one or more of the following: the RV mass to RV end-diastolic volume ratio, the RV mass to RV end-systolic volume ratio and the RV/LV mass ratio. The presence/absence and location of LGE in both the LV and RV were also assessed and recorded.

#### Estimation of PA stiffness

PA stiffness assessment included the following parameters^10^:

- PA elasticity (%) – the variation in luminal area during the cardiac cycle: (maximal area − minimal area/minimal area).
- PA distensibility (%/mmHg) – the variation in luminal area during the cardiac cycle for a given change in pressure: (PA elasticity/systolic PA pressure – diastolic PA pressure).
- PA compliance (mm^2^/mmHg) – absolute changes in luminal area for a given pressure variation: (maximal area – minimal area)/(PA pulse pressure).
- PA capacitance (mm^3^/mmHg) – the variation in volume (calculated by phase-contrast imaging) during the cardiac cycle for a given pressure variation: (RV stroke volume/(PA pulse pressure).
- Elastic modulus, reflects the changes in luminal areas as a consequence of pressure variations: (PA pulse pressure) × minimal area/(maximal area − minimal area).
- Stiffness index beta: the ln (systolic PAP/diastolic PAP)/PA elasticity.
- Pulse wave velocity (PWV) was calculated as described by Davies *et al*.^24^: PWV = √∆Q^2^/∆A^2^; Q is the flow and A is the area through the PA. The contours for the main PA cross-section were manually traced on the magnitude images, with the purpose of determining the maximal and minimal PA areas, as well as peak and average velocities of the PA flow using specialized software (Syngo.Via VB20A). The program then automatically calculated flow and velocity data. Main PA and AA diameters were measured at the level of the PA bifurcation.

### Statistical analysis

Data was tested for normality by the The Kolmogorov-Smirnov test. Data with normal distribution were expressed as mean ± standard deviation (SD), data with non-normal distribution – as median value ± interquartile range (IQR), and relative frequencies for categorical variables were expressed in percentages. Variables with normal distribution were processed using the Student’s t test, while variables with non-parametric distribution were analysed by the ANOVA test. Baseline patient characteristics, echocardiography, CMR measures, and invasive hemodynamics were compared between patient groups using Fisher’s exact test or a Kruskal–Wallis rank sum test. Spearman’s coefficient was used to analyse correlations between RV function, PA stiffness and PAP. Pending the results of univariable analysis, multivariable analysis was performed to assess independent correlations between PAP, RV remodelling parameters and PA stiffness indices. The relationship between the six-minute walk test distance and PA stiffness was tested by linear regression analysis. Areas under the curves (AUC) were calculated by receiver-operator characteristic (ROC) curve analysis with the purpose of determining the sensitivity, specificity, positive predictive value and negative predictive value of PA stiffness parameters for diagnosing PH, with 95% confidence interval (CI). Results were graphically represented. Event-free survival (time to first event) was generated by the Kaplan–Meier method and survival curves were compared by the log-rank test. A Cox hazards model was used to test the association between the composite end-point for adverse outcomes (cardiac death, Holter-documented ventricular tachycardia, hospitalization) and baseline co-variables, with results presented as hazard rations (HRs) with 95% CI. The statistical analysis was performed with MedCalc for Windows, version 16.1.2 (MedCalc Software, Mariakerke, Belgium); p values < 0.05 were considered statistically significant.

## Results

### Patient’s characteristics

In the test group, COPD was quantified as moderate in 11 patients, severe in 16 and very severe in 3 patients. Among all COPD patients included in the test group, 16 patients with PH were identified. Patients’ demographic data, laboratory findings, pulmonary function test parameters, echocardiography and RHC characteristics are summarized in Table 1. There were no statistically significant differences in terms of age, gender, body surface area or systemic artery pressures between the two groups. Patients from the test group had higher body mass indexes and were more likely to be active smokers, with impaired respiratory function and higher NT-proBNP levels. Significant differences occurred in diastolic function parameters such as the E/E’mitral ratio (determined by echocardiography) which was significantly higher in the test group. Poorer exercise capacity as assessed by the six-minute walk test was also evident in the test group.

**Table 1:** Demographics, laboratory, echocardiography and hemodynamic characteristics of patients included in the study.

*Variables*	Control group (n = 30)	Test group		p-Value*
		No PH (n = 14)	PH (n = 16)	
Age, years, mean (SD)	62 (8)	62 (6)	62 (6)	NS
Male gender, n (%)	18 (60)	10 (71.4)	9 (56.2)	NS
Body surface area, m^2^, mean (SD)	1.89 (0.2)	1.93 (0.2)	1.94 (0.2)	NS
Body-mass index, kg/m^2^, mean (SD)	27.3 (5.1)	33.4 (6.4)	30.0 (5.6)	0.004
Systolic artery pressure, mmHg, mean (SD)	122 (10.9)	140 (25.9)	141 (22.1)	NS
Diastolic artery pressure, mmHg, mean (SD)	76 (10.2)	82 (16.0)	84 (11.3)	NS
Heart rate, beats/min, mean (SD)	80 (14.4)	83 (15.8)	79 (14.1)	NS
Current smoking status, n (%)	9 (30)	13 (92.8)	14 (87.5)	<0.001
LV EDV by echography index, mL/m^2^, mean (SD)	64.9 (7.6)	59.5 (12.8)	60.5 (10.4)	<0.05
LV ESV by echography index, mL/m^2^, mean (SD)	23.1 (5.7)	23.4 (5.7)	24.5 (5.5)	0.001
LV ejection fraction, %, mean (SD)	68.2 (4.3)	59.5 (3.5)	59.2 (2.8)	<0.001
E/A mitral ratio, mean (SD)	1.2 (0.3)	1.0 (0.4)	1.5 (0.7)	NS
E/E’ mitral ratio, mean (SD)	7.8 (1.5)	8.7 (2.1)	10.2 (2.3)	0.001
Peak velocity of systolic tricuspid flow, cm/s, mean (SD)	186 (23)	276 (37)	402 (56)	0.001
Six-minute walk distance, m, mean (SD)	536 (67.7)	231 (73.7)	229 (77.3)	<0.001
FEV_1_, L, mean (SD)	2.7 (0.2)	1.6 (0.1)	1.6 (0.1)	<0.001
FCV, L, mean (SD)	3.4 (0.3)	2.6 (0.3)	2.6 (0.2)	<0.001
FEV_1_/FVC, %, mean (SD)	81 (4.9)	60 (5.4)	61 (5.9)	<0.001
PaO_2_, mmHg, median (IQR)	95 (93–98)	87 (74–98)	83 (68–90)	<0.001
PaCO_2_, mmHg, median (IQR)	41 (34–47)	50 (41–56)	53 (42–69)	<0.001
SaO_2_, %, median (IQR)	97 (94–99)	89 (82–91)	81 (68–91)	<0.001
eGFR, mL min^−1^ 1.73 m^−2^, median (IQR)	71 (68–105)	63 (56–98)	60 (49–82)	<0.05
NT-proBNP, pg/mL, median (IQR)	205 (83–311)	1340 (324–4389)	2260 (658–5200)	<0.001
Mean PAP, mmHg, median, (IQR)		20 (18–22)	42 (32–52)	<0.001
Systolic PAP, mmHg, median, (IQR)		37 (24–38)	60 (48–73)	<0.001
Diastolic PAP, mmHg, median, (IQR)		12 (11–19)	23 (19–31)	<0.001
Mean PCWP, mmHg, median (IQR)		8 (6–11)	10 (7–12)	<0.05
PVR index, Wood units/m^2^, median (IQR)		3.5 (2.4–6.2)	11.9 (4.8–14.4)	<0.001
Cardiac index, L/min/m^2^, median (IQR)		4.9 (2.6–7.2)	2.8 (2.3–5.5)	<0.05

Abbreviations: PH, pulmonary hypertension; LV, left ventricle; LVEDV, left ventricular end-diastolic volume; LVESV, left ventricular end-systolic volume; E/A mitral ratio, transmitral early diastolic velocity/transmitral late diastolic velocity; E/E’ mitral ratio, transmitral early diastolic velocity/septal mitral annular early diastolic tissue velocity; FEV1, forced expiratory volume in 1 second; FVC, forced vital capacity; PAP, pulmonary artery pressure; PCWP, pulmonary capillary wedge pressure; PVR, pulmonary vascular resistance; PaO_2_, arterial partial pressure of oxygen; PaCO_2_, arterial partial pressure of carbon dioxide; SaO_2_, arterial oxygen saturation; eGFR, Estimated Glomerular Filtration Rate; NT-proBNP, N-terminal pro-B type natriuretic peptide; IQR, interquartile range; SD, standard deviation.

^*^Represents statistical significance between groups.

During a mean follow-up period of 16 (6 to 38) months, 7 patients had majors cardiovascular events, as further described: cardiac death due to atrioventricular dissociation (n = 1); Holter ECG-documented ventricular tachyarrhythmia (n = 1), and hospitalization for heart failure (n = 5).

### CMR characteristics

CMR characteristics are detailed in Table 2. No significant differences in LV end-diastolic volumes have been recorded between the two groups. However, patients in the test group had lower LV ejection fraction. LV and RV wall mass indexes were significantly higher in the test group. Right chamber structural parameters (RA area, RV end-systolic volume, as well as the RV mass index) were higher in COPD patients, who also had poorer RV systolic function, assessed by the lower RV ejection fraction and TAPSE. LGE was evident in 7 test group patients (6 patients with PH and 1 patient without PH) and strictly distributed at mid-myocardial level in the basal segments of the RV septal insertion points. Phase-contrast examination revealed significantly increased PA maximal and minimal areas, PA elastic modulus, and stiffness indexes beta, while PA elasticity, distensibility, compliance and capacitance were diminished in test group patients (p < 0.001).

**Table 2:** CMR imaging characteristics in the study population.

Variables	Control group (n = 30)	Test group		p-Value*
		No PH (n = 14)	PH (n = 16)	
LV EDV index, mL/m^2^, mean (SD)	71.3 (12.4)	63.6 (10.2)	61.1 (10.6)	NS
LV ESV index, mL/m^2^, mean (SD)	25.1 (4.4)	27.2 (5.5)	26.9 (3.8)	0.01
LV ejection fraction, %, mean (SD)	64.1 (6.6)	59.5 (2.7)	57.5 (2.4)	<0.001
LV mass index, g/m^2^, mean (SD)	57.4 (17.9)	67.9 (8.1)	65.3 (13.0)	0.01
LA area, cm^2^/m^2^, mean (SD)	8.2 (3.2)	10.0 (2.2)	10.1 (2.7)	0.001
RV EDV index, mL/m^2^, mean (SD)	62.1 (12.1)	72.1 (13.3)	79.5 (10.1)	NS
RV ESV index, mL/m^2^, mean (SD)	23.2 (8.8)	30.6 (6.7)	41.1 (6.6)	<0.001
RV ejection fraction, %, mean (SD)	63.8 (9.4)	57.6 (3.4)	48.1 (5.1)	<0.001
RV mass index, g/m^2^, mean (SD)	22.6 (5.4)	33.4 (7.1)	41.6 (7.2)	<0.001
RA area, cm^2^/m^2^, mean (SD)	8.6 (2.3)	10.4 (2.5)	11.1 (1.4)	<0.001
TAPSE, mm, mean (SD)	20.7 (3.7)	15.4 (2.4)	15.7 (3.1)	<0.001
RV/LV mass ratio, mean (SD)	0.42 (0.15)	0.48 (0.07)	0.64 (0.08)	0.001
PA: AA diameter ratio	0.91 (0.13)	0.97 (0.13)	1.1 (0.14)	<0.001
Maximal PA area, cm^2^, mean (SD)	8.2 (1.4)	9.4 (1.5)	10.4 (1.9)	<0.001
Minimal PA area, cm^2^, mean (SD)	5.8 (1.1)	7.1 (1.2)	7.9 (1.3)	<0.001
PA elasticity, %, median (IQR)	41.5 (24–116)	38.5 (12–44)	29.5 (15–53)	0.001
PA distensibility, %/mmHg, median (IQR)	1.26 (0.39–3.60)	0.73 (0.25–2.39)	0.51 (0.23–1.02)	0.001
PA compliance, mm^2^/mmHg, median (IQR)	25.0 (10–50)	10.8 (3.6–46.0)	6.4 (3.7–11.1)	<0.001
PA elastic modulus, mmHg, median (IQR)	24 (14–63)	64 (11–190)	137 (82–216)	<0.001
PA stiffness index beta, median (IQR)	2.0 (1.1–3.4)	2.7 (0.53–8.1)	2.9 (2.1–7.4)	<0.001
PA capacitance, mm^3^/mmHg, median (IQR)	9.8 (4.4–19.6)	3.1 (1.7–13.4)	1.8 (1.4–3.0)	<0.001
PWV, m/s, mean (SD)	2.10 (0.7)	5.58 (1.4)	9.71 (2.2)	<0.001

Abbreviations: AA, ascending aorta; LA, left atrium; LV, left ventricle; LVEDV, left ventricular end-diastolic volume; LVESV, left ventricular end-systolic volume; PH, pulmonary hypertension; RA, right atrium; RV, right ventricle; RVEDV, right ventricular end-diastolic volume; RVESV, right ventricular end-systolic volume TAPSE, tricuspid annular plane systolic excursion; PA, pulmonary artery; PWV, pulse wave velocity; IQR, interquartile range; SD, standard deviation.

*Represents statistical significance between groups.

### Correlation Coefficients between PA Stiffness Parameters and Pulmonary Pressures and Resistance

Spearman’s coefficient analysis revealed significant associations between stiffness indices assessed by CMR, and PAP and PVR determined by RHC. Results are detailed in Table 3. PA distensibility, compliance and capacitance were inversely and strongly correlated to mean and systolic PAP, while associations to PVR index were only low to moderate.

**Table 3:** Correlation between RV function, PA Stiffness Parameters and Pulmonary Pressures/Resistancein Patients with COPD.

Variables	Mean PAP	Systolic PAP	PVR index
RV EDV index, mL/m^2^	−0.086	−0.089	0.189
RV ESV index, mL/m^2^	0.504*	0.517*	0.548*
RV ejection fraction, %	−0.653*	−0.670*	−0.691
RV mass index, g/m^2^	0.705*	0.703*	0.458
RA area, cm^2^/m^2^	0.373	0.387	0.196
RV/LV mass ratio	0.569*	0.554*	0.432
Maximal PA area, cm^2^	0.502*	0.592*	0.396
Minimal PA area, cm^2^	0.522*	0.608*	0.359
PA:AA diameter ratio	0.476*	0.452*	0.707*
PA elasticity, %	−0.328	−0.362	−0.480
PA distensibility, %/mmHg	−0.593*	−0.614*	−0.382
PA compliance, mm^2^/mmHg	−0.695*	−0.721*	−0.405
PA elastic modulus, mmHg	0.768*	0.787*	0.501*
PA stiffness index beta	0.315	0.351	−0.338
PA capacitance, mm^3^/mmHg	−0.714*	−0.756*	−0.746*
PWV, m/s	0.935*	0.934*	0.698*

Abbreviations: AA, ascending aorta; COPD, chronic obstructive pulmonary disease; LV, left ventricle; RA, right atrium; RV, right ventricle; RVEDV, right ventricular end-diastolic volume; RVESV, right ventricular end-systolic volume; PA, pulmonary artery; PAP, pulmonary artery pressure; PVR, pulmonary vascular resistance; PWV, pulse wave velocity; IQR, interquartile range; SD, standard deviation.

^*^Statistical significance: p < 0.0001.

PA elastic modulus was strongly and directly correlated to mean PAP and systolic PAP, as was minimal PA area. The PA:AA diameter ratio was also strongly correlated to the PVR index, although associations to mean and systolic PAP were only moderate. The strongest associations emerged between PWV and mean and systolic PAP, with reasonably strong associations to the PVR index.

PWV was negatively correlated to PA distensibility (r = −0.537; p < 0.0001), compliance (r = −0.766; p < 0.0001) and capacitance (r = −0.867; p < 0.0001), and positively correlated to the elastic modulus (r = 0.776; p < 0.0001) and stiffness index beta (r = 0.401; p < 0.001). The strongest associations occurred with PA capacitance and the elastic modulus.

### Relationship between Pulmonary Artery Stiffness Indices and the Six-Minute Walk Test Distance

**Figure 1:**
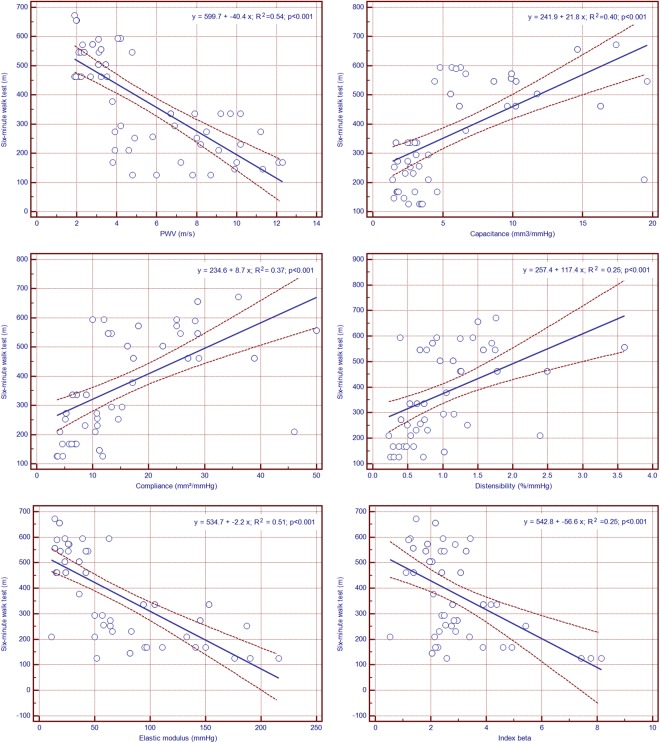
Scatter plots showing linear relationships between six-minute walk test distances and pulmonary artery stiffness indices.

Positive correlations were obvious between the six-minute walk test distances and PA distensibility, compliance and capacitance. In addition to that, the six-minute walk test distance was negatively correlated to the PWV, elastic modulus and index beta after linear regression analysis (Fig. 1).

**Figure 2:**
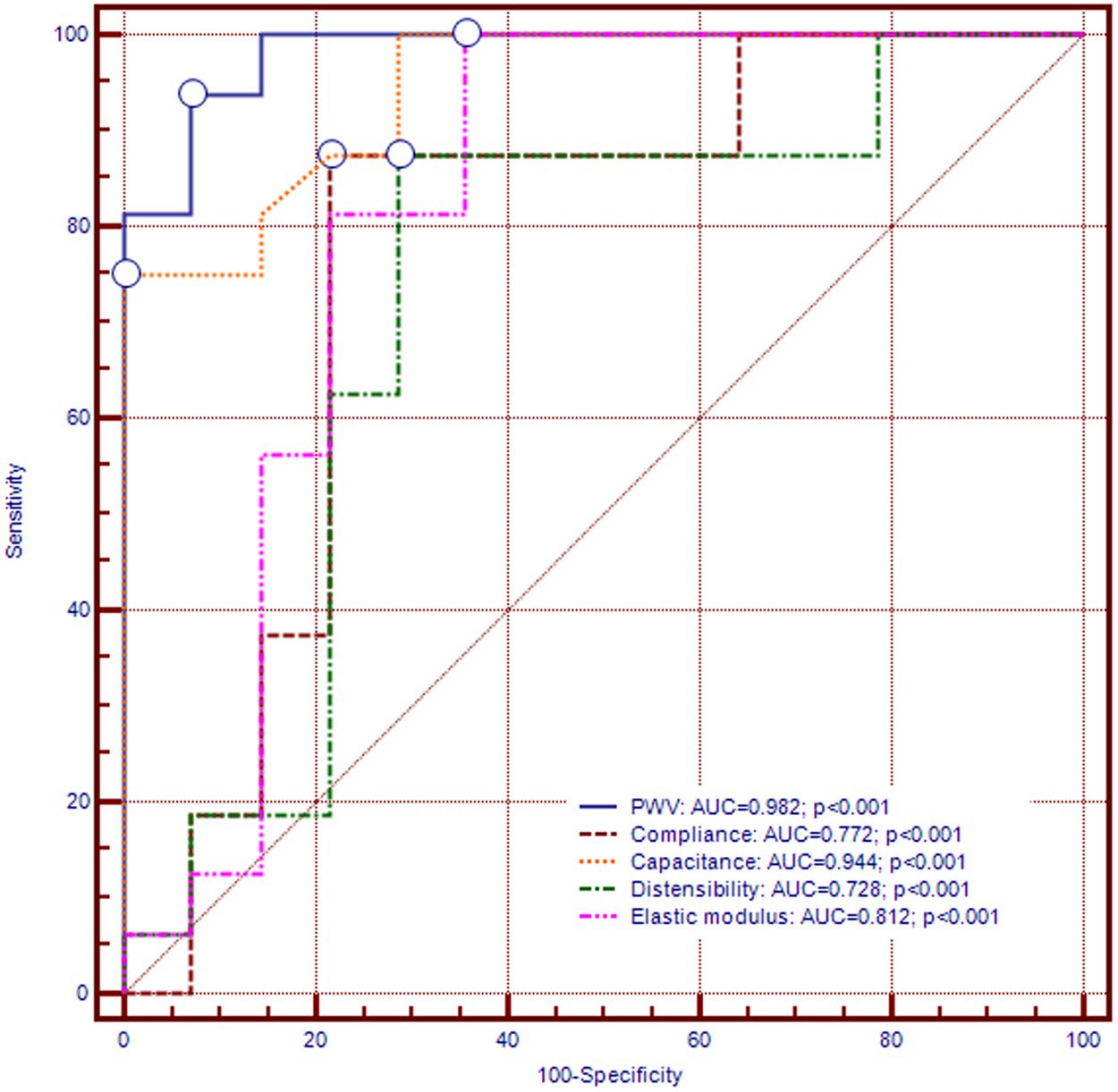
Receiver operator characteristic curve highlighting the diagnostic ability of pulmonary artery stiffness indices for identifying pulmonary hypertension.

**Figure 3:**
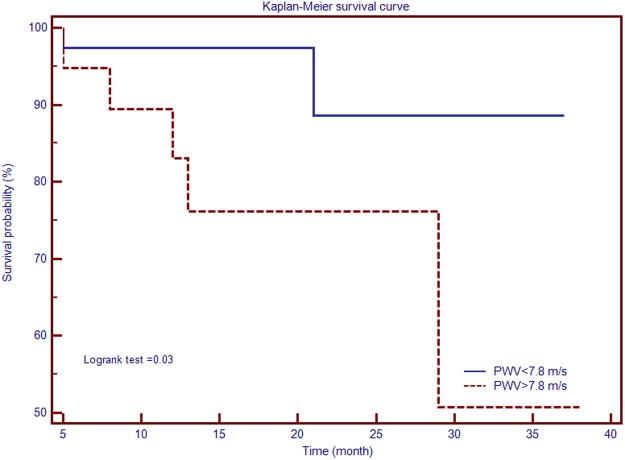
Kaplan–Meier curves yielding the value of pulsed wave velocity for predicting event-free survival in patients with secondary pulmonary hypertension due to chronic obstructive pulmonary disease.

Multivariable analysis was used to explore the association between RV and PA morphological and functional parameters, as assessed by CMR, and mean and systolic PAP, in the presence of some potentially confounding factors, such as age or gender. To that purpose, four different models were constructed: model A included age, gender and RV mass index; model B – age, gender and PA elastic modulus; model C – age, gender and PWV; model D – age, gender, RV mass index, PA elastic modulus and PWV. Results are detailed in Table 4. Among all studied parameters, PWV performed best, being correlated to both mean and systolic PAP independently of age, gender, RV mass index, and PA elastic modulus (R^2^ = 0.89, p < 0.001; and R^2^ = 0.89, p < 0.001, respectively). The predictive abilities of different PA stiffness indices for detecting PH as evaluated by ROC curve analysis have been compared (Fig. 2). For PWV, ROC curve analysis showed an AUC of 0.982 (95%CI: 0.948 to 0.999, p < 0.0001). The optimal cut-off value for PWV was established at 7.8 m/s, yielding 93.5% sensibility, 92.8% specificity, 93.7% positive predictive value and 92.9% negative predictive value for identifying PH. Associations between CMR-derived PWV as marker of PA stiffness and the overall occurrence of major cardiovascular events were assessed by Kaplan–Meier analysis. Pending this analysis, patients who exhibited higher PWV were more likely to experience MACEs by comparison with patients with low PWV [HR 4.75 (95% CI 1.00 to 22.59), p = 0.03] (Fig. 3).

**Table 4:** Relationships between PAP and pulmonary artery stiffness indices.

	Mean PAP			Systolic PAP		
	β	Individual (p)	Overall R² (p)	β	Individual (p)	Overall R² (p)
**Model A**						
Age	0.025 ± 0.164	0.875	0.52 (<0.001)	0.153 ± 0.129	0.249	0.51 (<0.001)
Gender	4.591 ± 2.508	0.071		1.169 ± 1.552	0.457	
RV mass index	0.851 ± 0.112	0.0001		1.250 ± 0.167	<0.0001	
**Model B**						
Age	−0.032 ± 0.152	0.983	0.59 (<0.001)	−0.017± 0.216	0.936	0.62 (<0.001)
Gender	1.7826 ± 2.350	0.450		1.160 ± 3.337	0.729	
PA elastic modulus	1.759 ± 0.020	<0.0001		0.267 ± 0.028	<0.0001	
**Model C**						
Age	−0.023 ± 0.083	0.783	0.87 (<0.001)	−0.280 ± 0.179	0.122	0.87 (<0.001)
Gender	1.159 ± 1.290	0.372		4.187 ± 6.486	0.520	
PWV	3.758 ± 0.192	<0.0001		5.596 ± 0.283	<0.0001	
**Model D**						
Age	−0.015 ± 0.080	0.845	0.89 (<0.001)	−0.009 ± 0.115	0.932	0.89 (<0.001)
Gender	0.922 ± 1.232	0.425		0.613 ± 1.775	0.972	
RV mass index	0.057 ± 0.051	0.217		0.077 ± 0.074	0.297	
PA elastic modulus	0.037 ± 0.015	0.020		0.071 ± 0.022	0.002	
PWV	3.009 ± 0.349	<0.0001		4.261 ± 0.504	<0.0001	

Abbreviations:: regression coefficient β is given for mean PAP and systolic PAP. Adjustment models: model A: age, gender; RVmass index; model B: age, gender; minimal PA area; model C: age, gender; PWV; model D: age, gender; RV mass index, PA elastic modulus and PWV. Abbreviations: RV, right ventricle; PA, pulmonary artery; PAP, pulmonary artery pressure; PWV, pulse wave velocity.

### Sample size estimation

For assuring a 95% confidence, and 80% power of the study and based on the difference in MACE between groups, we have calculated a minimum required sample size of 40 patients in the test group for testing the ability of cardic MRI derived parameters to identify patients at risk for MACEs.

## Discussion

Currently, data regarding PWV measurements by CMR in patients with COPD are scarce. In our study, significant inverse correlations occurred between CMR-measured PWV and the indices for compliance and distensibility of the pulmonary vasculature, thus indicating that CMR could be used for early non-invasive identification of PH. As shown in previous studies, the assessment of PWV in major arteries provides crucial information regarding PA stiffness^23^. Interestingly, in our study, PWV performed best among all PA stiffness parameters in identifying PH, as ROC curve analysis yielded reasonable performance indices.

In addition to that, the results of our research highlighted the presence of right chamber remodelling assessed by echocardiography and CMR in patients with secondary PH due to COPD. Right chamber dimensions and RV mass were considerably elevated in test group patients by comparison with healthy volunteers, which was expected, considering the fact that the RV responds to elevated pulmonary pressures by progressive hypertrophy, followed by dilatation^25^. A previous CMR-based research on patients with COPD and no or mild hypoxemia revealed that RV mass was increased in test patients by comparison with healthy volunteers despite normal RV systolic function, suggesting that RV hypertrophy is the earliest sign of RV pressure overload, occurring as an adaptation process^26^. Another study comparing healthy volunteers to COPD patients with or without PH supported the same hypothesis; the study showed that changes suggesting RV hypertrophy were more obvious in COPD patients without PH vs. healthy controls, with little increase in patients with COPD and PH^27^. RV dilatation is also an essential component of RV remodelling. In our study, CMR measurements of RV dimensions, as well as RV end-diastolic volumes were significantly more elevated in COPD patients. Previous research has shown that larger RV volumes measured by CMR were an independent predictor of higher overall and cardiovascular mortality^28^.

Patients in our study were only admitted to the test group provided they had evidence of PH. In our research, CMR examination revealed impaired RV systolic function, as assessed by TAPSE, the RV ejection fraction and RV stroke volume in COPD patients by comparison with healthy volunteers. RV stroke volume reduction was also obvious in a previous study by Roeleveld *et al*. who also showed improvement of this parameter after specific vasodilator treatment^29^, while van Wolferen *et al*. demonstrated that reduced RV stroke volume was an independent predictor of increased mortality and treatment failure^30^.

In a study by Kjaergaard *et al*., diminished TAPSE was shown to be highly predictive of increased mortality in patients with COPD and PH^31^; similar results were obtained by Forfia *et al*. who demonstrated significant correlations between TAPSE and right chamber remodelling, as well as other indices of impaired RV systolic function, and showed that patients with PH and decreased TAPSE had lower survival^32^.

Our CMR-based examinations also showed significant differences in PA stiffness indices between test group patients and healthy volunteers. In addition to that, statistical analysis revealed important correlations between PA stiffness indices, such as PA elasticity, distensibility, capacitance, compliance, elastic modulus, stiffness indices alpha and beta, and RV remodelling and function parameters, such as RV ejection fraction, RV mass indexed, RV stroke volume indexed, TAPSE, and RV end-systolic volume; these findings are consistent with results from other studies^33^. Interestingly, there was barely any correlation to RV end-diastolic volumes, an observation which also emerged from the study of Stevens *et al*.^19^. These results may suggest that RV dilatation with no impairment in RV systolic function is less likely to be accompanied by significantly increased PA stiffness. In fact, RV systolic impairment may be a consequence of pressure overload triggered by persistent severely increased PA stiffness, since a previous study showed that PA stiffness indices became impaired before significant increases in resting systolic PAP^10^, and may, therefore, have a significant contribution to disease progression. Accordingly, we intended to establish correlations between systolic PAP and PA stiffness indices; the strongest linear correlations were obvious between systolic PAP and PA distensibility, compliance, capacitance and elastic modulus, which is consistent with the results of previous studies in which either CMR^10^ or intravascular ultrasound were used for determining pulmonary stiffness^34,35^.

Interestingly, low PWV values at CMR were shown to predict survival, a novel discovery, undocumented, to our knowledge in previous research. However, results should be interpreted with caution, considering the low number of patients, and implicitly the low number of individuals who experienced MACEs.

Indeed, the main limitations of the current study are related to the rather small number of participants. We aimed to diminish the impact of this limitation on our results by carefully selecting the study population using well established criteria and following a standard detailed examination protocol. Also, the study needs to be continued until the minimal estimated sample size is reached.

CMR examinations were carefully performed, yielding good quality images. 2D-velocity-encoded CMR allowed accurate non-invasive measurements of aortic blood flow parameters with good temporal and spatial resolutions. Although 4D phase-contrast CMR was not available for the assessment of the patients in this study, the limited temporal and spatial resolutions that have been reported for such techniques^36^ rendered this examination superfluous. Further research on a larger number of patients may help consolidate the results of the current study.

## Conclusions

CMR phase-contrast imaging may provide an extensive and accurate assessment of right chambers remodelling and PA stiffness in patients with COPD. In the future, it may become a valuable non-invasive tool for quantifying PH, provided that its value is further confirmed by more extensive studies. PA stiffness assessment by CMR seems to be promising in terms of early identification of pulmonary vascular disease, which may result in earlier diagnosis and treatment onset, thus diminishing arterial remodeling progression. Moreover, CMR-derived PA stiffness indices, particularly PWV, may be useful for predicting MACEs. However, our research is only hypothesis generating and larger studies are necessary to confirm these findings.
